# Learning flat optics for extended depth of field microscopy imaging

**DOI:** 10.1515/nanoph-2023-0321

**Published:** 2023-08-02

**Authors:** Ipek Anil Atalay Appak, Erdem Sahin, Christine Guillemot, Humeyra Caglayan

**Affiliations:** Faculty of Engineering and Natural Science, Photonics, Tampere University, 33720 Tampere, Finland; Faculty of Information Technology and Communication Sciences, Tampere University, 33720 Tampere, Finland; INRIA Rennes – Bretagne Atlantique, Rennes, France

**Keywords:** diffractive optics, end-to-end learning, extended depth of field, metasurfaces, microscopy imaging

## Abstract

Conventional microscopy systems have limited depth of field, which often necessitates depth scanning techniques hindered by light scattering. Various techniques have been developed to address this challenge, but they have limited extended depth of field (EDOF) capabilities. To overcome this challenge, this study proposes an end-to-end optimization framework for building a computational EDOF microscope that combines a 4f microscopy optical setup incorporating learned optics at the Fourier plane and a post-processing deblurring neural network. Utilizing the end-to-end differentiable model, we present a systematic design methodology for computational EDOF microscopy based on the specific visualization requirements of the sample under examination. In particular, we demonstrate that the metasurface optics provides key advantages for extreme EDOF imaging conditions, where the extended DOF range is well beyond what is demonstrated in state of the art, achieving superior EDOF performance.

## 1 Introduction

A conventional imaging system can produce sharp images for objects within the depth of field (DOF), which is the range around the focused depth of the scene. The DOF coverage is inversely proportional to the numerical aperture (NA), i.e., a smaller NA leads to a larger DOF. Although specific applications may require smaller DOFs, a larger DOF is often preferable to obtain sharp images of objects at varying depths. In particular, microscopy requires high NA objectives to capture precise details surrounding the focused depth of an object. However, due to the shallow DOF of high NA imaging systems, microscopic imaging systems often use depth scanning techniques to cover the entire depth range of interest, typically much larger than the imaging system DOF [1]. Depth scanning techniques are often insufficient due to the light scattering from objects outside the intended image plane, resulting in artifacts or severe noise. Acquiring cross-sectional data using this approach requires sweeping a focused point across the entire sample, which inherently imposes temporal limitations on the frame rate and prevents snapshot acquisition. To address these challenges, various techniques have been developed, such as decoupled illumination and detection in light-sheet microscopy [2], dynamic remote focusing [3, 4], and spatial and spectral multiplexing [5, 6]. Despite their potential advantages, these methods often necessitate a specialized and intricate optical configuration, which may render them both costly and difficult to implement in microscopy applications. In addition, computational approaches such as Fourier ptychographic microscopy demonstrated extended depth of field (EDOF) imaging capabilities [7]. However, this method relies on image reconstruction that assumes a thin sample illuminated by oblique plane waves, rendering it unsuitable for clinical fluorescence imaging applications.

The integration of wavefront encoding with computational reconstruction methods offers a cost-effective and efficient approach to improve EDOF imaging performance. Specifically, computational reconstruction methods, such as deep learning-based approaches and iterative optimization algorithms, have demonstrated substantial advancements in enhancing the EDOF imaging performance by effectively mitigating defocus blur, noise, and artifacts [8–12]. These methods leverage the optimization of both optical components and the reconstruction algorithm, enabling the system to enhance the EDOF performance effectively. End-to-end optimization frameworks, which jointly optimize the optical design and the associated reconstruction algorithms, have emerged as a powerful tool for addressing the EDOF challenge. This approach allows the system to learn and adapt to specific imaging requirements, thereby improving overall performance and enabling better control over trade-offs in resolution, noise, and depth range [8]. Wavefront coding involves the use of a phase element, such as a diffractive optical element (DOE) or a free-form refractive lens, placed at the aperture plane [13–19]. The main objective of EDOF wavefront coding is to achieve a depth-invariant point spread function (PSF) while preserving information at all spatial frequencies. The research presented in [10] employs DOEs to realize EDOF microscopy across 200 μm DOF range, a methodology closely aligned with our study. However, DOE wavefront coding displays restricted EDOF capabilities, a limitation that may be attributed to constraints within the space-bandwidth product (SBP). This factor determines the information content captured by the imaging system. A detailed comparison with such method, called DeepDOF, is elaborated in Section 3.

In comparison, metasurfaces, which are ultra-thin meta-optics composed of subwavelength nano-antennas, offer increased design flexibility and a superior SBP compared to DOEs [20, 21]. These structures facilitate precise control over phase, amplitude, and polarization of light at the nanoscale, allowing almost arbitrary modification of the complex optical functions on a thin, planar device. The advantages of metasurfaces can be attributed to the rich modal characteristics of meta-optical scatterers, enabling multifunctional capabilities beyond traditional DOEs, encompassing polarization, frequency, and angle multiplexing [22–25]. Consequently, metasurfaces exhibit a greater potential for addressing the EDOF microscopy challenge more effectively than conventional DOEs, with researchers having already leveraged their benefits in various applications such as flat optics for imaging [26, 27], polarization control [28], and holography [29]. Despite the promising potential of meta-optics, current metasurface imaging methodologies demonstrate limited EDOF imaging capabilities. The EDOF range for any given imaging system can be quantified using the defocus coefficient, wherein a larger EDOF corresponds to a higher defocus coefficient. Details regarding these defocus coefficients and their relationship with the depth of field range and optical parameters are comprehensively covered in Section 2.1. Currently, most methods are designed to accommodate systems with a maximum defocus coefficient limited to around 75, with some using mechanical displacement as a strategy to extend imaging capability [30–32]. Although the narrow defocus ranges may be adequate for certain applications, it is comparatively limited in broader scientific and industrial contexts. As such, there remains a need for more flexible and versatile imaging solutions that can accommodate a broader depth range without sacrificing image quality. In contrast, the study in [33], addresses a problem characterized by a maximum defocus coefficient comparable to ours, valued at around 245. However, the demonstrated image quality is considerably lower compared to the results achieved in our research. It is worth noting that other metasurface-based imaging implementations exist, which are capable of encoding spatial, spectral, and polarization information while maintaining satisfactory imaging performance [34, 35]. Moreover, computational metasurface designs that yield a large field-of-view for full-color metasurface operation, without significant degradation of imaging performance, have been reported [21].

In this study, we propose an end-to-end optimization framework designed for acquiring high-resolution images across an extensive DOF range within a microscopy system. The optics and post-processing algorithm are modeled as parts of the end-to-end differentiable computational image acquisition system, allowing for simultaneously optimizing both components. Our computational EDOF microscope employs a hybrid approach that combines a 4f microscopy optical setup with a learned wavefront modulating optical element at the Fourier plane. We explore metasurfaces and (conventional) DOEs for implementing such novel design modulation. The encoded image acquired at the sensor is post-processed by a convolutional neural network (CNN) that implements deblurring to achieve an EDOF image of the specimen. The optimization procedure involves tuning of critical parameters within two primary components of the system: the optical component, characterized by the phase modulation function, and the image reconstruction component, realized through the deblurring convolutional neural network (D-CNN). This process is facilitated by an end-to-end learning methodology that utilizes a dataset of sharp images to steer the optimization. This methodology emphasizes the importance of carefully outlining sampling requirements for various depth-of-field targets in order to achieve optimal imaging results. As a result, our systematic design methodology significantly outperforms existing state-of-the-art EDOF imaging techniques in terms of image quality and depth range.

## 2 Methods

**Figure 1:**
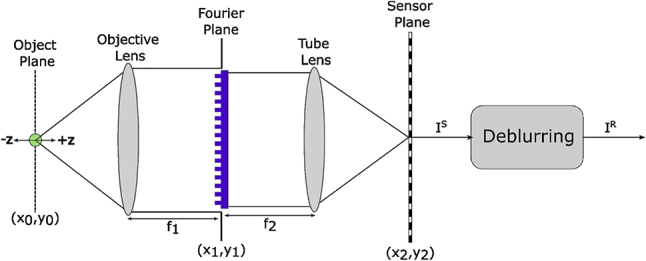
A 4f system computational EDOF microscope model that combines the optical module, sensor, and D-CNN.

The EDOF microscopy problem addresses the difficulty of capturing high-resolution images across an extensive DOF range within a microscopy system. The objective of resolving this problem is to improve the imaging performance of the system by expanding its DOF, allowing the acquisition of sharp images over a larger depth range without necessitating mechanical focus adjustments. Figure 1 presents the proposed 4f system designed to address the EDOF microscopy problem, which consists of an optical module, a sensor, and a subsequent deblurring module for post-processing. The optical module employs a 4f imaging configuration that incorporates a phase coding mask at the aperture position. This mask, an optical element crafted to modulate incident light, introduces phase shifts across various regions through a transparent material with spatially varying geometry. The purpose of using a phase coding mask is to manipulate the optical wavefront, enabling a specific PSF or other desired properties within the optical system. In addressing this problem, the 4f system components remain fixed while optimizing the EDOF microscopy imaging system by learning the spatial distribution of the phase coding mask and the D-CNN weights for the targeted DOF ranges. Consequently, the objective is to achieve a defocus-invariant PSF within the optical system, resulting in improved EDOF microscopy imaging performance.

**Figure 2:**
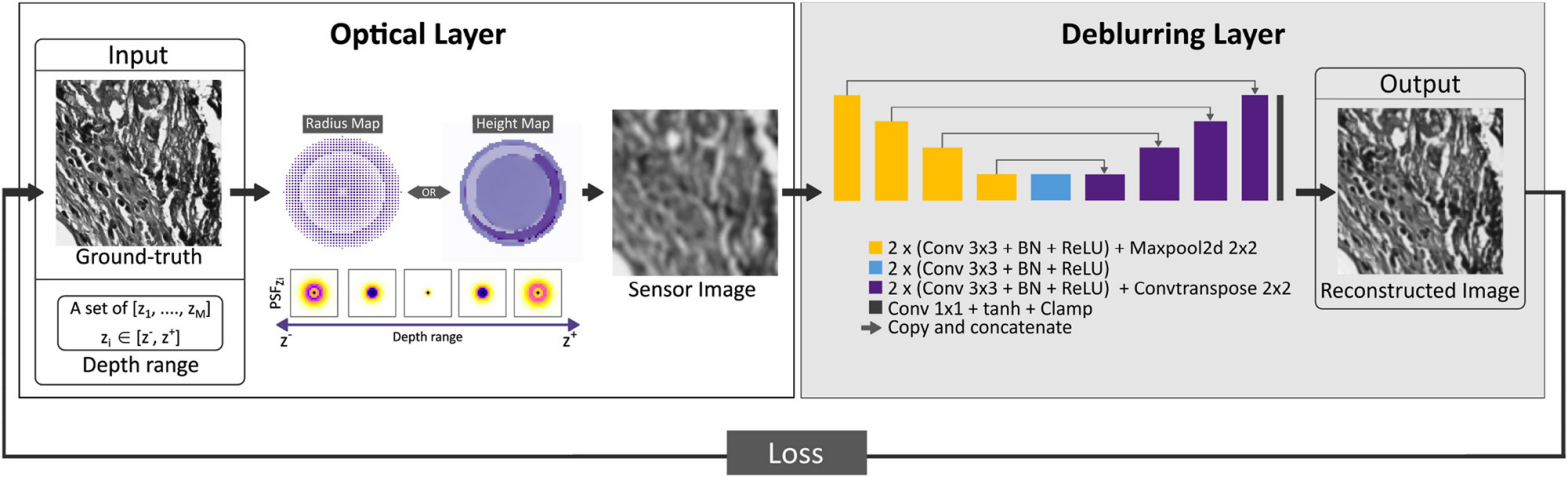
Overall representation of the end-to-end learning framework for joint optimization of the phase coding mask and the D-CNN. The end-to-end framework consists of an optical layer that simulates image formation for the learned optics, and the deblurring layer employs U-Net architecture to reconstruct in-focus images within the desired DOF range. The optical element can be realized via a metalens, parametrized by the radius map, or a DOE, parametrized by the height map. The choice of representation relies upon the maximum Δ*s* calculated for the intended depth range.

Figure 2 depicts the proposed end-to-end learning procedure for the EDOF microscopy problem. This process accepts two inputs as high-resolution image patches and the predetermined depth range. The architecture of the framework consists of two main components: an optical layer and a D-CNN layer. During the training phase, the sensor image is generated through the optical layer, which involves simulating the image acquisition process based on depth value and the input image. The resulting sensor image is then fed into the D-CNN, which estimates the sharp, deblurred image as its output. The backpropagation algorithm is used to update the spatial phase distribution of the phase coding mask and the weights of the D-CNN at each iteration of the learning procedure, thus optimizing the parameters of the EDOF microscope in an end-to-end manner. The optimized phase coding mask is realized as a meta-optic element (metalens) or DOE, depending on the spatial phase distribution and sampling. After training is completed for the targeted DOF range, the defocus-invariant optical element and D-CNN model are obtained in the 4f system computational EDOF microscope model (Figure 2). Further details regarding the optical and D-CNN layers are provided below.

### 2.1 Optical layer

The optical layer is a computational module that operates based on the principles of wave optics and performs sensor image formation. In the following, we discuss sensor image formation by employing wave optics in particular and characterize the parameters of the EDOF microscope for the targeted DOF range for a 4f shift-invariant imaging system as illustrated in Figure 1. Here, the specimen is illuminated by a monochromatic, spatially incoherent light source. The spatial coordinates in the Fourier and sensor planes are denoted as (*x*_1_, *y*_1_) and (*x*_2_, *y*_2_), respectively. A transparent biological specimen in the system can be represented as a stack of 2D images corresponding to a fixed scene depth where a slice of such a stack corresponds to *I*_*z*_(*x*_2_, *y*_2_) at the scene depth *z*. The contribution of such a slice to the sensor image, $I_{z}^{s}\left(x_{2},y_{2}\right)$ , is determined by convolution with the depth-dependent PSF, *h*_*z*_(*x*_2_, *y*_2_):

(1) $$I_{z}^{s}\left(x_{2},y_{2}\right)=I_{z}\left(x_{2},y_{2}\right)\;*\;h_{z}\left(x_{2},y_{2}\right).$$

The final image captured at the sensor, *I* ^*s*^(*x*_2_, *y*_2_), is then determined by integrating $I_{z}^{s}\left(x_{2},y_{2}\right)$ over all depth values possible in the scene, considering

(2) $$I^{s}\left(x_{2},y_{2}\right)=\int I_{z}^{s}\left(x_{2},y_{2}\right)\;dz+\eta_{s},$$

where *η*_*s*_ is a sensor noise. In our simulations, we consider the noise as a zero-mean Gaussian model with $\eta_{s}\approx N\left(0,\sigma_{s}^{2}\right)$ , where *σ*_*s*_ represents the standard deviation of the Gaussian noise. It is important to note that while we have assumed Gaussian noise for our experiments, the proposed method can be easily adapted to handle other noise models, such as Poisson distributed noise, by adjusting the noise assumption within the optimization process. Considering Eqs. 1 and 2, the recovery of a sharp image directly depends on *h*_*z*_(*x*_2_, *y*_2_). The PSF on the sensor plane can be modeled using Fourier optics as the square of the Fourier transform of the generalized pupil function:

(3) $$h_{z}\left(x_{2},y_{2}\right)=|F\left\{P\left(x_{1},y_{1}\right)\right\}{|}^{2},$$

where *F*{.} denotes the Fourier transform operator, and *P*(*x*_1_, *y*_1_) is the pupil function, which describes the relative amplitude and phase changes of the wavefront at the Fourier plane:

(4) $$P\left(x_{1},y_{1}\right)=A\left(x_{1},y_{1}\right){\text{e}}^{\text{i}\phi \left(x_{1},y_{1}\right)}.$$

The pupil can be modulated using a phase coding mask introducing a phase term *ϕ* ^*M*^(*x*_1_, *y*_1_) to enhance the defocus invariance of the PSF in the targeted DOF range. The resultant phase term becomes:

(5) $$\phi_{z}\left(x_{1},y_{1}\right)=\phi_{z}^{DF}\left(x_{1},y_{1}\right)+\phi^{M}\left(x_{1},y_{1}\right).$$

The defocus aberration due to the mismatch between in-focus depth *z*_0_ and the actual depth *z* of a scene point is

(6) $$\phi_{z}^{DF}\left(x_{1},y_{1}\right)=\psi_{z}\frac{x_{1}^{2}+y_{1}^{2}}{r^{2}},$$

where $\psi_{z}=\frac{\pi }{\lambda }\left(\frac{1}{z}-\frac{1}{z_{0}}\right)r^{2}$ is the defocus coefficient and *r* is the radius of the pupil. To represent all spatial frequencies *k*_*x*_, *k*_*y*_ supported by the objective lens on the pupil plane, the pupil size must be chosen using

(7) $$r\geq \frac{f_{1}}{k}\sqrt{k_{x}^{2}+k_{y}^{2}},$$

where *f*_1_ is the focal length of the tube lens, and *k* is the wave number. The frequency support of the PSF, in particular, determines the reconstruction quality. To avoid aliasing due to undersampling of the defocused pupil function, which would otherwise result in a miscalculated PSF, the minimum sampling rate of the simulated phase coding mask must be determined. The sampling rate Δ*s* for a given depth range should satisfy [11]

(8) $$\Delta s\leq \frac{r\pi }{8\psi_{\max}},$$

where $\psi_{\max}=\max\left(\left|\psi_{z^{-}}|,|\psi_{z^{+}}\right|\right)$ , the maximum defocus value within the scene. At the same time, we use such a relation as a guide in choosing the search space for the phase modulation function of the optical element, matching its degree of freedom with the complexity of the EDOF imaging problem at hand. This is observed to have a significant help in the end-to-end learning process, i.e., its convergence to the optimal solution. The optimized optical element can be realized as a metalens or DOE according to the calculated maximum Δ*s* for the targeted depth range. The metalens and DOE are parameterized by a radius map and a height map, respectively, details of which are provided in the following sections.

During training, the optical element is optimized to minimize the impact of the defocus phase and ensure depth-invariant PSFs through the utilization of learned phase coding mask elements. For each iteration within the forward pass, this optimization is achieved by calculating Eq. (3) and optimizing the *ϕ* ^*M*^(*x*_1_, *y*_1_) phase modulation term. For both the DOE and the metalens, the amplitude *A*(*x*_1_, *y*_1_) of the pupil function is kept constant within the aperture diameter and is modeled as a circular function. The camera parameters *f*_1_, *r*, and Δ*s* are predefined as optical parameters. *r* and Δ*s* is calculated for the selected objective lens and targeted depth range using Eqs. (7) and (8), respectively.

For the DOE design, the phase modulation can be controlled through unit cell height. Specifically, the phase shift is given by the equation:

(9) $$\phi^{M}\left(x_{1},y_{1}\right)=k\left(n-1\right)h\left(x_{1},y_{1}\right),$$

where *n* denotes the refractive index of the DOE material which is 1.5, specifically at the design wavelength of 550 nm within the green spectrum. The DOE is assumed to be lossless; therefore the amplitude is kept fixed as the circular function within the aperture diameter.

**Figure 3:**
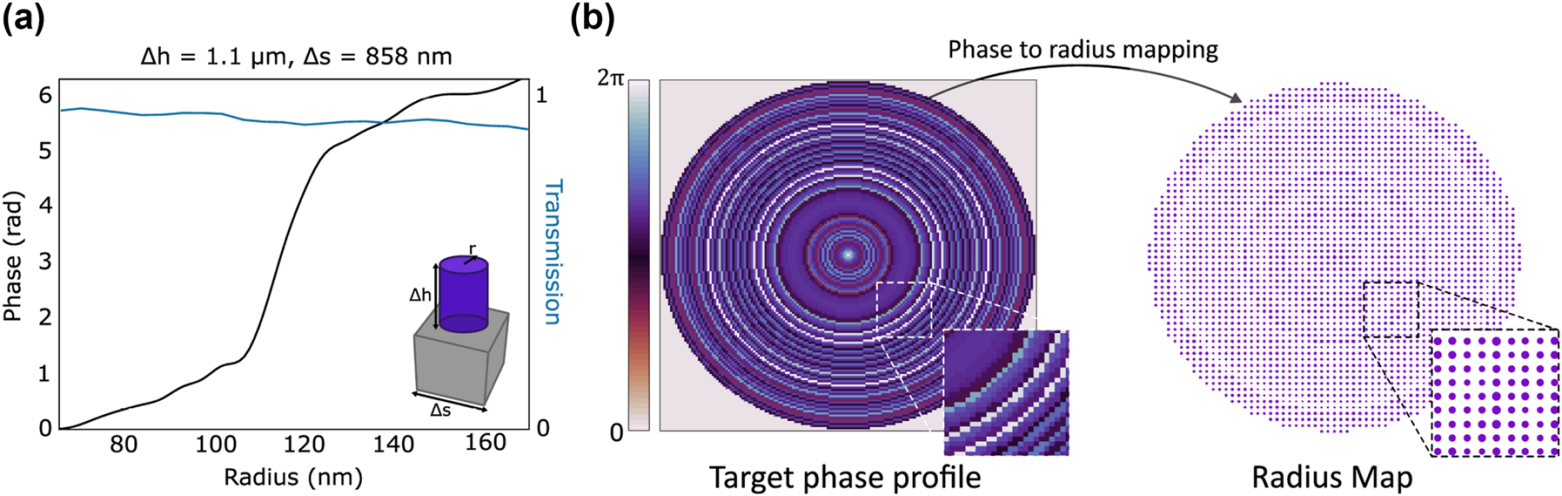
Design and simulation of EDOF imaging metalenses. The metalenses are made up of GaN nanopillars with a refractive index of 2.42, where the thickness (Δ*h*), sampling (Δ*s*), and radius (Δ*r*) are the design parameters. (a) Simulation of the nanopillars’ transmission amplitude and phase response via FDTD. (b) Illustration of the full metalens design, transforming the spatial phase profile into a spatial radius (of nanopillar) distribution with respect to the target phase.

For the metalens design, phase accumulation is achieved through the waveguiding effect [36], whereby the height of the nanopillars is selected to provide 2*π* phase coverage across a range of radii. While the smallest possible diameter is primarily limited by fabrication constraints, the largest diameter is 50 nm smaller than Δ*s*, as set by Eq. (8). To ensure high efficiency, the nanopillar height Δ*h* is optimized at the 550 nm design wavelength. The phase and transmission responses are simulated via a finite-difference time-domain (FDTD) analysis, which involves varying the radius of the gallium nitride (GaN) nanopillar on a sapphire glass substrate. As depicted in Figure 3(a), the analysis results in full phase coverage (0–2*π*) with high transmission (overall greater than 83 %) for 550 nm.

Irrespective of the desired target phase, designing a metalens involves converting a spatial phase profile into a corresponding spatial radius distribution. A resulting *ϕ* ^*M*^(*x*_1_, *y*_1_) phase profile, generated from the end-to-end framework, is illustrated in Figure 3(b). This profile is then translated into the full metalens, taking into account the simulated phase and radius response. This approach enables the design of highly precise and effective metalens that meets the desired phase requirements.

### 2.2 Deblurring CNN

The D-CNN module shown in Figure 2 utilizes the sensor output (*I* ^*s*^) from the optical layer as its input. Although many network architectures exist for this problem, we chose the well-known U-Net [37] as it is widely used in biomedical imaging for image reconstruction [10, 38, 39]. In short, the U-Net implementation has an encoder and decoder architecture with 23 convolution layers and 32 to 512 feature channels. At each step of the encoder stage, the input underwent two 3 × 3 convolution layers, a rectified linear unit (ReLU), and batch normalization (BN). Subsequently, the feature map is downscaled by a 2 × 2 max-pooling operation. Likewise, following two 3 × 3 convolution layers that incorporated ReLU and BN at each iteration of the decoder, a 2 × 2 transposed convolution operator upsampled the feature map. At the final layer, a 1 × 1 convolution is used to map each 32-component feature vector to the desired number of classes, which is 1 in the current case. Moreover, a hyperbolic tangent (tanh) activation is utilized to map the output to the range of [−1, 1]. The residual image is then incorporated by addition to the sensor output. The Clamp layer further processes the resulting image to constrain the data from 0 to 1.

For memory efficiency, U-Net takes input as 256 × 256 blurry pixel images corresponding to different depths and outputs the enhanced images at each iteration. It should be noted that to avoid border distortions during convolution with the PSF, the patched image and PSF are convolved and subsequently cropped to 256 × 256 after convolution. Once the network is trained, it can process images with dimensions that are multiples of 16 in width and height.

### 2.3 End-to-end learning

The end-to-end framework is trained via an image dataset provided by [10]. The dataset consists of a diverse array of microscopic fluorescent images of proflavin-stained oral cancer resections, histopathology images of healthy and cancerous tissues from the Cancer Genome Atlas (TCGA), and natural images from the National Digital Science and Technology Research Institute’s (INRIA) Holiday dataset. This diverse selection ensures a broad range of feature scales within the dataset. The dataset contains a total of 1800 grayscale images, 1000 × 1000 pixels each, and includes 600 images of each type. The images are randomly assigned to training, validation, and testing datasets, with 1500, 150, and 150 images, respectively. During training, the images are randomly cropped and enhanced with rotation, flipping, and brightness adjustments for data augmentation. Throughout the training and validation stages, at each iteration, a random image patch is selected from the corresponding dataset and assigned to *M* depth positions *z*_*i*_ ∈ (*z*_1_, …, *z*_*M*_) that are uniformly distributed (in diopters) within the boundaries of the targeted scene depth, as illustrated in Figure 2. During a forward pass, the loss function corresponding to each depth is computed and subsequently summed and averaged over the number of selected depths. The calculation of the loss function for each depth involves sequentially processing the image patches assigned to the chosen *z*_*i*_. In this study, we limited *m* to five depth positions to reduce the computational complexity. Upon experimentation, an increase in the number of depth positions did not yield a significant improvement in imaging performance.

The loss function used in the framework is the root mean squared error (RMSE) calculated for each depth and each pixel of the reconstructed image stack, compared to the respective pixel in the ground-truth image, with the results subsequently averaged across the number of depths:

(10) $$L_{\text{RMSE}}=\frac{1}{M}\overset{M}{\underset{i=1}{\sum }}\frac{1}{\sqrt{N}}\|I-I^{R}{\|}_{2},$$

where *N* is the number of pixels. As the input experiences blurring at varying defocus values throughout the depth, the framework intrinsically adapted the optics to achieve a defocus-insensitive PSF. Consequently, no explicit cost function is required to maintain PSF similarity within the designated depth range.

The PyTorch package is used to implement the framework, and stochastic gradient descent with the Adam optimizer [40] is employed for optimization. The learning rates for the Adam optimizer are selected empirically as 1*e* − 7 for the optical layer and 1*e* − 4 for the D-CNN. During end-to-end framework training, a two-step training process is used, where the initial step is training the U-Net with fixed optics. After the convergence, joint training of the optical rendering and D-CNN is performed to achieve optimal performance. Regarding computational requirements, we used two Tesla P100-PCIE-12GB GPUs for training. Depending on the complexity of the problem, which is dictated by the number of required parameters, the training duration varies. The most parameter-intensive problem necessitated a training period of approximately 148 h and 33 min, whereas the least demanding scenario required a comparatively shorter duration of around 6 h and 41 min.

## 3 Results

Our design is aimed to mitigate the trade-off between DOF and spatial resolution for varying target DOFs by experimenting with different objective lenses within the 4f imaging setup, as illustrated in Figure 1. This trade-off is mathematically expressed as follows:

(11) $$DOF\propto \frac{\lambda }{{NA}^{2}}\propto \frac{{resolution}^{2}}{\lambda }.$$

This demonstrates that the spatial resolution is higher for a shallower DOF range; therefore, a shallower DOF range optimization problem is comparatively less challenging to solve. The complexity of the problem, however, is affiliated with the selected lenses and the desired DOF range. It is quantified and expressed using the defocus coefficient (*ψ*_*z*_) and presented alongside the system parameters for different simulations in Table 1.

**Table 1:** Test results and calculated system parameters for the selected lenses.

Objective lens	NA	DOF	*r* (mm)	Δ*s*(μm)	PSNR	SSIM	*ψ*_max_
RMS4X-PF^a^	0.13	±100 μm	3.72	740	38.19	0.98	0.97
RMS4X-PF	0.13	±1 mm	3.72	73	30.11	0.88	9.877
RMS20X-PF	0.40	±100 μm	2.87	38	29.95	0.90	14.69
Mitutoyo50X	0.42	±100 μm	1.07	20	30.84	0.88	10.48
Mitutoyo50X^a^	0.42	±1 mm	1.07	1.54	29.28	0.89	136.24
Mitutoyo50X^a^	0.42	±1.5 mm	1.07	0.858	27.98	0.89	245.54

^a^Used as the optimized design for the targeted DOF range.

Table 1 demonstrates that solving the EDOF problem for a higher NA lens system is considerably more challenging task, where the sampling requirements, determined by Eq. (8), become more demanding due to the broader bandwidth of the defocused pupil function, necessitating finer sampling for accurate results. Such a case, thus, advocates the metalens to address the underlying stricter sampling requirement. Conversely, for the same EDOF range, using a low NA lens-based system with a learned DOE can yield a satisfactory solution. However, it should be noted that the final magnification would be significantly lower in such a system. Therefore, the lens selection should be based on the requirements of the sample to be visualized. To the best of our knowledge, certain biological specimens, such as the developing embryo, exhibit a diameter that increases from approximately 200 μm to nearly 3 mm [41]. As such, we have selected to target DOF ranges of 200 μm, 2 mm, and 3 mm for our study.

To further support the results, we compare the proposed algorithm with two existing methods. Initially, a cubic mask is adopted as the conventional method for DOF extension [13], which modulates the phase as

(12) $$\phi^{M}\left(x_{1},y_{1}\right)=\mathrm{mod}\left[\frac{\alpha }{N^{3}}\left(x_{1}^{3}+y_{1}^{3}\right),2\pi \right],|x|<\frac{N}{2},|y|<\frac{N}{2},$$

where *α* determines the number of 2*π* transitions. In this case, a fixed cubic phase mask is combined with a U-Net and subsequently trained to perform deblurring for all EDOF targets. To select an appropriate mask for each DOF range, the modulation transfer function (MTF) is evaluated across five different depths by tuning *α*, to be insensitive to defocus and represent all spatial frequencies. *α* values of 6*π*, 200*π*, and 360*π* are selected for the DOF ranges of 200 μm, 2 mm, and 3 mm, respectively.

Additionally, the DeepDOF algorithm proposed by Jin et al. [10] is adopted as a more recent advanced EDOF method based on the end-to-end learning framework and Zernike basis (*Z*_*n*_) representation. Notably, the paper does not provide a relationship between the choice of the number of Zernike polynomials and the EDOF range. To align the optical setup parameters with our methodology and ascertain the appropriate number of parameters (*n*) for each depth range, we calculated the Fourier transform of the *Z*_*n*_ and found the bandwidth of the resultant signal. Considering the Nyquist theorem, we calculated the sampling rate for the *Z*_*n*_. Based on these calculations, we selected the first 11, 1100, and 2000 *Z*_*n*_ for DOF targets of 200 μm, 2 mm, and 3 mm, respectively.

**Figure 4:**
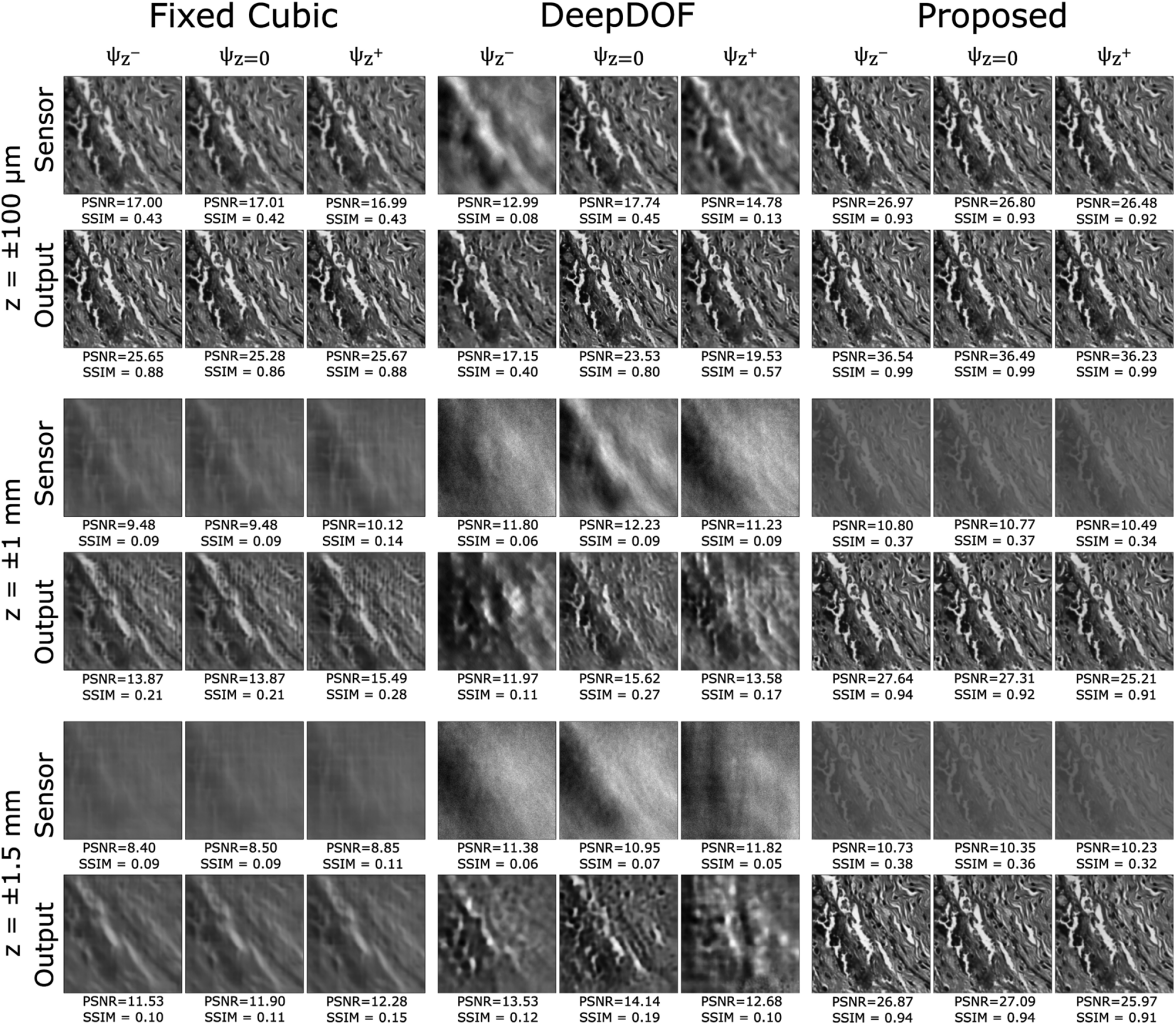
Simulated performance of a cubic-mask-enabled computational 4f system, DeepDOF model, computational EDOF microscope, optimized for various target depths. For all DOFs, our method shows superior performance.

We present a quantitative analysis of the proposed method in comparison with existing approaches by analyzing the performance metrics, specifically peak signal-to-noise ratios (PSNRs) and structural similarities (SSIMs). For each test setup, the PSNR and SSIM values are derived and depicted in Figure 4. Additionally, the mean PSNR and SSIM values are calculated for all images within the 150 test images of the dataset and presented in Table 2. To calculate the average values, we assign each test image to predetermined depths, uniformly distributed throughout the scene depth range. Subsequently, the PSNR and SSIM values are averaged.

**Table 2:** Quantitative analysis of methods for EDOF problem. It should be noted that all the methods are simulated using the same system parameters that are computed for each DOF range. Refer to Table 1 for system parameters.

Framework configuration	DOF	PSNR	SSIM
Fixed cubic + U-Net	±100 μm	34.99	0.92
	±1 mm	23.6	0.62
	±1.5 mm	21.86	0.58
DeepDOF [10]	±100 μm	28.90, 28.85^a^	0.80, 0.81^a^
	±1 mm	20.67	0.55
	±1.5 mm	21.14	0.58
Our method	±100 μm	38.19	0.98
	±1 mm	29.28	0.89
	±1.5 mm	27.98	0.89

^a^Used first 55 Zernike polynomials.

As demonstrated in Table 2, the proposed method outperforms existing approaches in terms of PSNR and SSIM values, both for the sample image shown in Figure 4 and the mean values across the entire test dataset. In particular, the D-CNN alone, as inferred from the fixed Cubic mask and U-Net simulations, is inadequate. Upon increasing the target DOF, the image becomes excessively blurry at the sensor level, and the U-Net cannot recover the images effectively. Comparable results are visible in the DeepDOF approach, which is linked to the sub-sampling of the frequency domain that arises from using the Zernike basis representation. It is important to acknowledge that [10] is optimized using 55 Zernike basis, for a target DOF of 200 μm. The starred result in Table 2 displays the outcomes obtained when employing 55 Zernike basis. In this case, we retain the same setup parameters as in our other simulations, only modifying the number of Zernike basis utilized to represent the height map in the method, resulting in a higher sampling than necessary. The results demonstrate that increasing the sampling does not improve the performance, thus confirming that our calculated setup parameters sufficiently address the system complexity. Additionally, in our U-Net implementation, different from [10], we adapted a modified U-Net architecture for the deblurring layer. The main difference resides in the final layer of our U-Net architecture, where we incorporated the residual image and applied the clamp function, enhancing both the accuracy and efficiency of the deblurring process.

**Figure 5:**
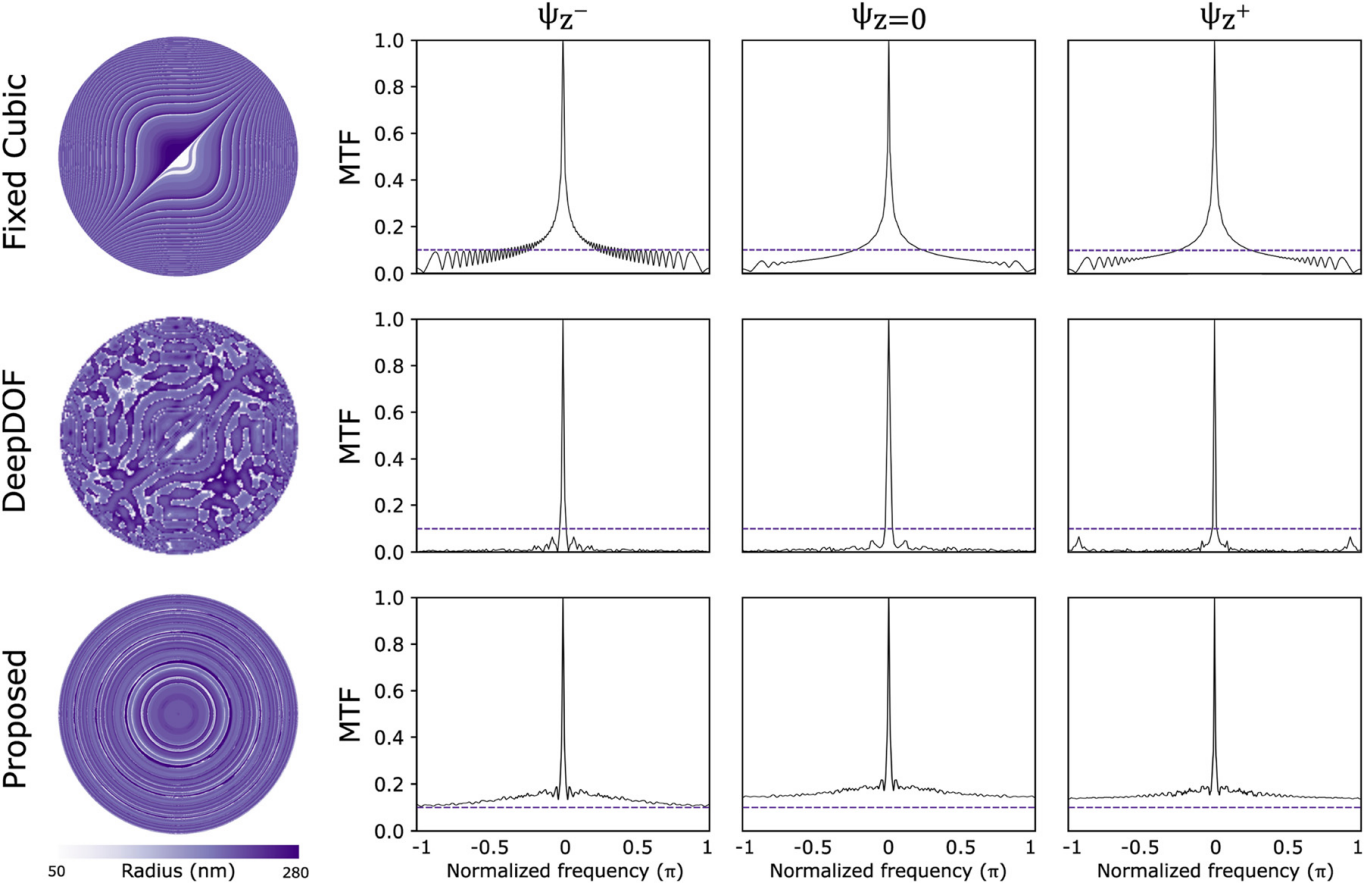
The optimized metalens radius maps, as well as the MTFs at three distinct depths, are presented for the existing and proposed methods, encompassing the maximum defocus values within the 3 mm DOF scene and the in-focus depth.

An alternative method for evaluating EDOF performance involves examining the characteristics of MTFs across various depths, with MTFs representing the magnitudes of the Fourier transforms corresponding to their associated PSFs [12]. To facilitate efficient depth-agnostic deblurring throughout the whole depth range of interest, MTF pass-bands should be as wide as possible to recover features at various spatial frequencies while maintaining a high degree of similarity among themselves. Figure 5 presents the final metasurface designs for the 3 mm DOF scenario, as well as the MTFs at three distinct depths. The depth dependency of the MTFs in the proposed method is decreased compared to both the DeepDOF and Fixed cubic cases. The MTFs of the proposed method display greater similarity among themselves and avoid crossing zero, ensuring the preservation of information during recovery. This similarity is attributed to depth-agnostic deblurring assisting MTFs to remain consistent across all targeted depths. To compare the frequency support of each method, we defined a threshold of 0.1 and examined the MTF magnitude across the frequency spectrum and depths, which formally determines the maximum spatial resolution transmitted through the optics. The MTFs of the proposed method resulted in broadband coverage across the entire spectrum, illustrating that even at higher spatial frequencies, MTFs do not approach zero. This finding corresponds to the clear visibility of small structures. Conversely, at lower spatial frequencies, the MTF is closer to 1, representing the ability to clearly visualize large structures. Indeed, as demonstrated in the other methods, all MTFs can clearly visualize large structures, but for smaller structures, quality is inadequate.

### 3.1 Fabrication error analysis

**Figure 6:**
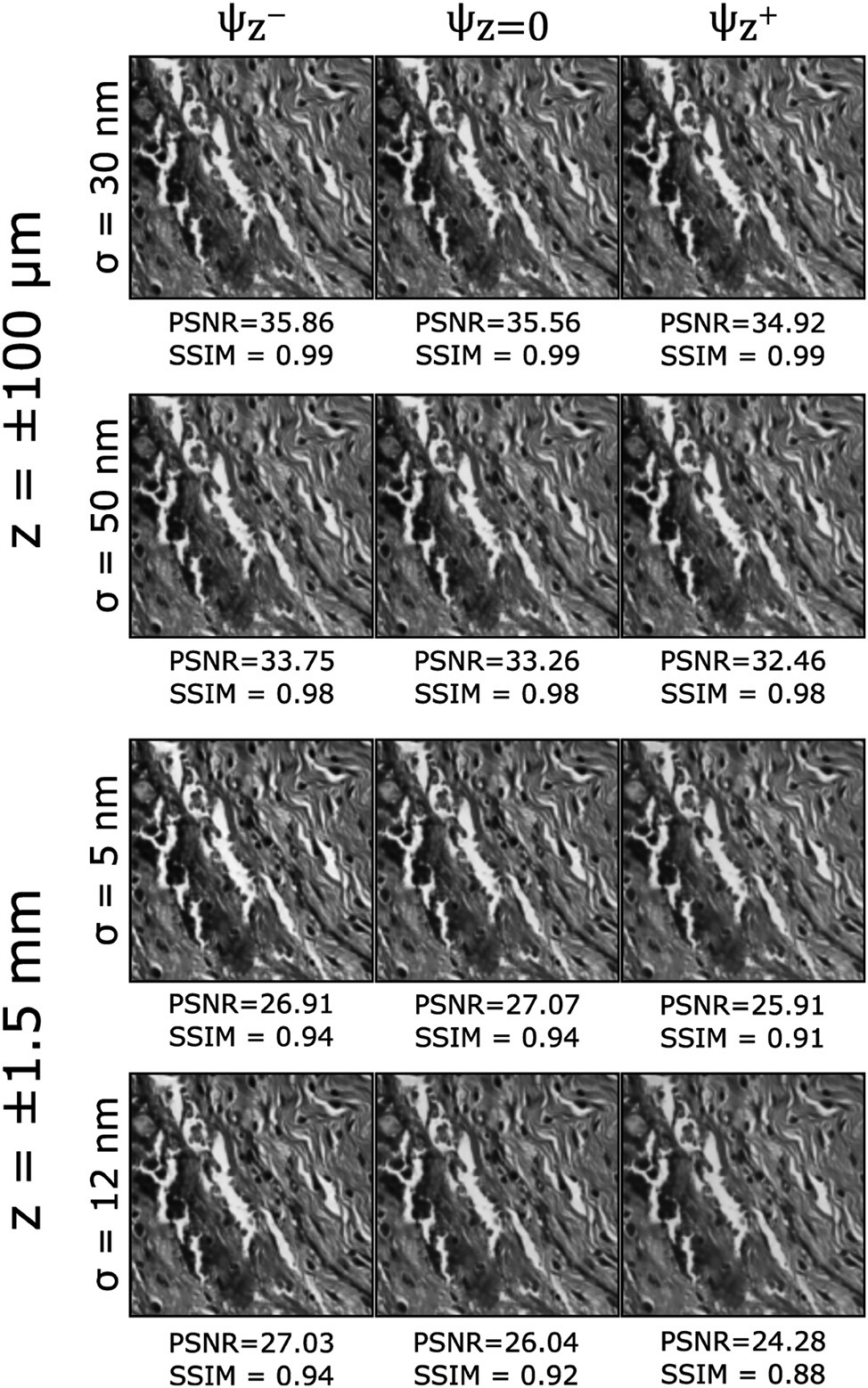
Reconstruction results for increasing radius and height-map fabrication error levels.

The robustness of the proposed EDOF microscope model is tested considering various levels of inaccuracies for the possible fabrication errors of metasurface and DOE. In particular, the effects of such fabrication inaccuracies are modeled by introducing random Gaussian-distributed noise with a standard deviation *σ*_*r*_, to the optimized optical elements, as the radius and DOE height error during the test stage. The tested metasurface radius deviation levels are *σ*_*r*_ = 5 nm, 12 nm, while the DOE height deviation is assumed as *σ*_*h*_ = 30 nm, 50 nm. Figure 6 presents the outcomes for the various EDOF configurations. As it can be inferred from the figure, the post-processing method performs adequately for lower fabrication error levels but exhibits limited robustness in the presence of higher error margins, leading to a noticeable reduction in image quality, particularly at the boundaries of the targeted EDOF. The robustness of the algorithm can be increased by incorporating fabrication error boundaries during training or retraining the D-CNN following the fabrication of optical components. Additionally, the implementation of advanced denoising algorithms can contribute to the enhancement of the results.

## 4 Future work and conclusion

In conclusion, we implemented an end-to-end optimization framework that effectively addresses the physical limitations inherent in the 4f system EDOF microscope. Our approach offers two primary advantages over existing methods. Firstly, the incorporation of metasurface optics within our system has facilitated the achievement of the most extensive EDOF range reported in the literature thus far. In extreme EDOF scenarios, specifically when employing a higher NA lens, meta-optics offer a distinct advantage due to their increased design flexibility and superior SBP. Secondly, our method effectively minimizes defocus by systematically incorporating relevant optical system parameters throughout the optimization process. The results obtained from our simulations demonstrate that our proposed technique outperforms the current state-of-the-art methods in EDOF microscopy imaging, delivering consistently high performance across a broad range of EDOF values.

In future work, we aim to investigate the co-design of optics and post-processing for broadband EDOF imaging. Moreover, we aim to explore the application of our approach in addressing the challenges associated with light field microscopy. Our next objective is fabricating a selected metasurface, followed by a comprehensive evaluation of its performance within the context of the 4f system setup. We believe that these research directions will contribute significantly to the ongoing advancement of microscopy imaging, ultimately leading to more sophisticated and versatile optical systems.
